# Evaluation of Four Incisions Used For Radical Neck Dissection- A Comparative Study

**DOI:** 10.31557/APJCP.2019.20.2.575

**Published:** 2019

**Authors:** Satadru Roy, Vikram Shetty, Vishwanath Sherigar, Padmaraj Hegde, Rajendra Prasad

**Affiliations:** 1 *Deprtment of Oral and Maxillofacial Surgery, A.B.Shetty Memorial Institute of Dental Sciences, *; 2 *Department of Oncology, Justice K.S.Hegde Charitable Hospital, Mangalore. *

**Keywords:** Oral cancer, head and neck cancer, oral squamous cell carcinoma, radical neck dissection

## Abstract

**Objective:**

To evaluate the four commonly used incisions for Radical Neck Dissection on the basis of certain defined parameters.

**Patients and Methods:**

The investigators designed and implemented a prospective comparative study composed of patients with oral squamous cell carcinoma. The predictor variable was time taken to raise and close the flaps, accessibility to the neck lymph nodes, injury to vital structures and scar cosmesis followed up to a period of three months. Descriptive statistics were computed.

**Results:**

The sample was composed of 40 patients grouped as follows: Macfee Incision (=10 patients), Modified Macfee Incision (=10 patients), Modified Schobinger Incision (=10 patients) and Reverse Hockey Stick Incision (=10 patients). Group A, consisiting of the patients with Macfee Incision, took the least time to close among all the groups ( Mean= 32.60 minutes) while Group C (patients with Modified Schobinger Incision) required the most time for closure ( Mean= 51.90 minutes). The Modified Schobinger Incision provided best exposure to neck node levels. The Macfee Incision was found to have the best scar cosmesis among the four incisions.

**Conclusion:**

The results of this study suggest that Modified Schobinger Incision is the preferred incision for adequate access to neck lymphatics while Macfee Incision was found to provide the best scar cosmesis.

## Introduction

Oral cancer treatment often involves the removal of affected lymph node groups in the neck. The extension of the disease to the neck lymph nodes makes the treatment challenging because of close proximity of carotid arteries, internal jugular vein and cranial nerves (Tubach et al., 2013). Surgical incision is a critical element of operative planning. Therefore, surgical incisions should be planned appropriately to improve oncologic resection without compromising functional or esthetic outcomes. 

Neck incisions have been described in the literature for more than a century. In 1906, Crile’s landmark paper described a Y-shaped incision, which was used for decades, the modifications of which are in common use today (Crile, 1987; Goswamy and Murthy, 2014). Because neck incision gained acceptance as part of general treatment of oral cancer, various skin incisions were introduced to provide a suitable approach to the cervical lymphatics (Macfee, 1960). Since the 1950s, single transverse incisions have been regularly used. The use of these incisions has engendered the development of the Macfee Incision, which comprises of two horizontal parallel incisions (Macfee, 1960). Single linear incisions are cosmetically most appropriate because they lie in the relaxed skin tension lines. Transverse Incisions can be adapted to any usual methods of neck dissection. The creation of acute angles or the convergence and crossover of incisions is conveniently avoided in this incision. However, while describing the Macfee incision with two parallel horizontal incisions, Macfee commented that the access was limited (Macfee, 1960). Thus, Macfee incision may lead to compromise in disease clearance. In view of the limitations encountered in Macfee Incision, a modification of the Macfee Incision was proposed by Kudva et al., (2014), who altered the upper limb of the incision. The modification provides excellent exposure of all neck node levels and a narrower bridge, thus facilitating an easier dissection of spinal accessory nerve. The reversed-Hockey Stick Incision was first described by Schobinger as a long anterior skin flap for radical Neck Dissection and was extended to block the resection of oral cancer by Babcock and Conley and Dissanayaka (Schobinger, 1957; Babcock and Conley, 1966; Dissanayaka, 1990; Omura et al., 1999). It allowed for an inferiorly based single cervical skin flap. Omura et al., (1999) did a comparative study on the use of Hockey Stick Incision and Reverse Hockey Stick Incision for neck dissection. The results of the comparative study revealed that the Reverse Hockey Stick Incision was particularly suitable for access to all five levels of lymph nodes and provided optimum exposure of the oral cavity through the transverse limb of the neck incision. 

No specific incision or a combination of incisions has received universal acceptance. The need for selection of an appropriate neck incision that would provide adequate access to neck nodes to ensure optimum disease clearance without compromising the vascularity is paramount. Hence, this study compared the results of these commonly used incisions for radical neck dissection on the basis of their accessibility to operation site, duration of operation, post-operative wound healing, damage to vital structures and scar cosmesis developing a standardized outcome, which will form the basis of the selection of the most suitable incision for a given case of neck metastasis. 

## Materials and Methods

Patients who reported to the Department of Oral and Maxillofacial Surgery, A.B. Shetty Memorial Institute of Dental Sciences and Leela Narayan Shetty Memorial Cancer Institute, Mangalore were enrolled in the study.

*Inclusion criteria*

• Patients who had undergone modified radical/radical neck dissection for a proven case of squamous cell carcinoma of the oral cavity and were 20–65 years old were included in the study.

*Exclusion criteria*

• Patients who had undergone preoperative radiotherapy or chemotherapy, exhibited tumors with clinical or radiological skin involvement, and refused to provide written consent were excluded.

Forty patients were randomly divided into four groups of ten patients each-

**Figure 1:**
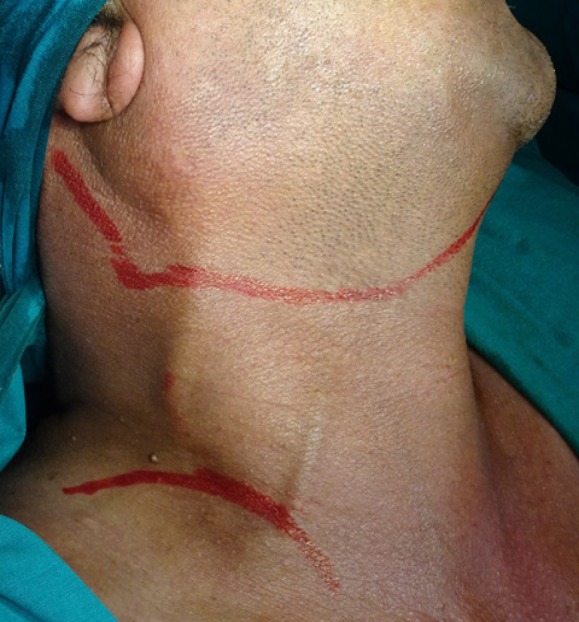
Incision Marking For Macfee Incision

**Figure 2:**
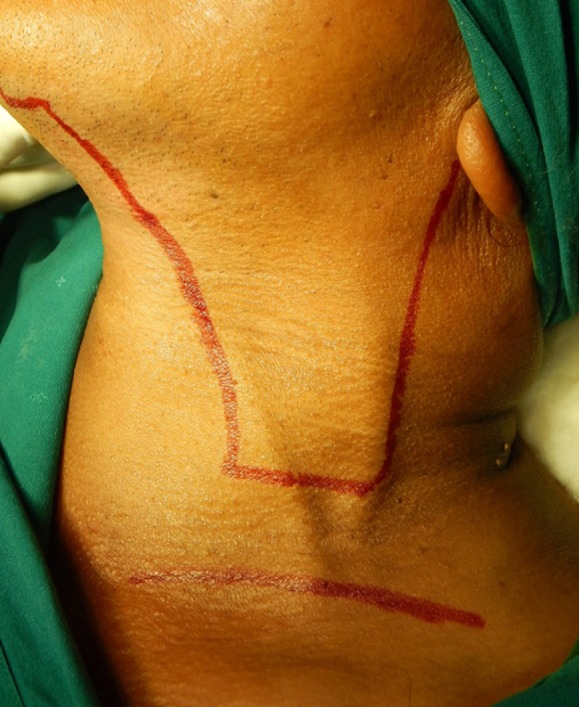
Incision Marking for Modified Macfee Incision

**Figure 3:**
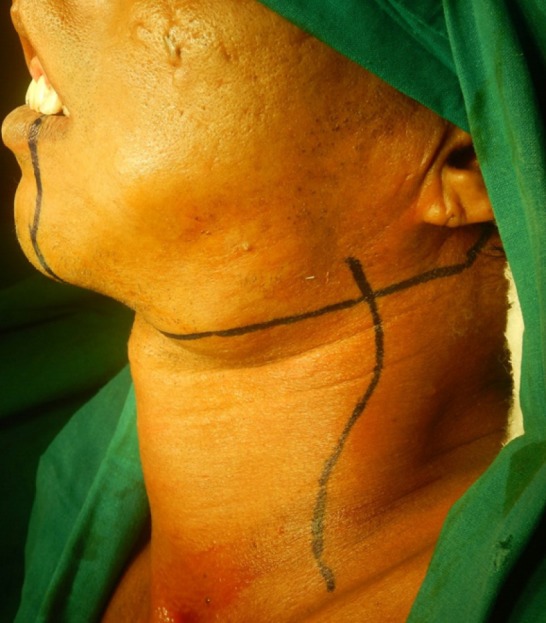
Incision Marking For Modified Schobinger Incision

**Figure 4:**
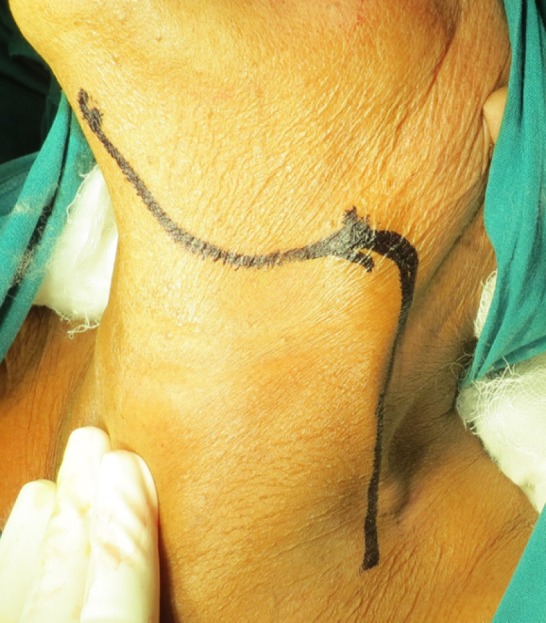
Incision Marking For Reverse Hockey Stick Incision

1. Group A: Macfee Incision (Figure 1)

2. Group B: Modified Macfee Incision (Figure 2).

3. Group C: Modified Schobinger Incision (Figure 3)

4. Group D: Reverse Hockey Stick Incision(Figure 4).

An ethical clearance was obtained for the study from the Institutional Ethical Review Committee. The guidelines of the Declaration of Helsinki were followed and appropriate informed patient consent was obtained for the study. 

*Surgical Technique*

The patients were anesthetized through nasotracheal or orotracheal intubation. Surgical skin preparation of the face, neck, chest, temporal region, forearm, upper limb, and lower limb was performed using betadine depending on the type of reconstruction planned, and sterile draping of the patient was performed. The incision was marked; lignocaine (2%) for local infiltration was administered along the marking. The incision was made using No. 15 BP Blade. The platysmal layer was identified and raised to perform neck dissection. Either radical neck dissection or modified radical neck dissection was performed depending on the extent and involvement of vital structures. Reconstruction, if performed, was performed using various flaps such as the pectoralis major myocutaneous flap, temporalis flap, radial forearm flap, and skin graft. 

Intraoperative time required to raise the flap, accessibility to the neck, damage to the internal jugular vein, and the time required to close the flap were analyzed. The time required to raise the flap was calculated from the start of the incision until the point of starting the neck dissection. The time required for closure of the flap was calculated from the time of approximation of the flap to the completion of the last skin suture.

The incision and wound healing were postoperatively assessed at discharge and at monthly follow-ups for 3 months. Scar cosmesis was evaluated using the Stony Brook Scar Evaluation Scale (Table 1).

*Statistical Analysis*

Descriptive statistics, means, and standard deviations (SDs) were calculated for all variables. One-way analysis of variance (ANOVA) and Bonferroni multiple comparison tests were used to compare the means of the four groups. A value of P < 0.05 was considered statistically significant. Microsoft Excel and the SPSS software version 22 were used for statistical analysis.

## Results

The study included 40 patients (26 men and 14 women) who were 35–65 years old, and the mean age of the patients was 51.50 (SD: ±8.36) years. 

**Table 1:** Stony Brook Scar Evaluation Scale

Scar Category	Points
WIDTH	
>2 mm	0
<2 mm	1
HEIGHT	
Elevated/Depressed with respect tourrounding skin	0
Flat	1
Colour	
Darker than surrounding skin	0
Same colour or lighter than surrounding skin	1
Hatch marks/Suture Marks	
Present	0
Absent	1
Overall appearance	
Poor	0
Good	1

**Table 2:** Bonferroni Multiple Comparison to Assess the Time Taken to Raise the Flap (in Minutes) among the Four Groups

(I) group	(J) group	Mean Difference (I-J)	P	95% Confidence Interval	
				Lower Bound	Upper Bound
Group A	Group B	10.400*	<0.001	6.64	14.16
	Group C	5.900*	0.001	2.14	9.66
	Group D	12.500*	<0.001	8.74	16.26
Group B	Group A	-10.400*	<0.001	-14.16	-6.64
	Group C	-4.500*	0.012	-8.26	-0.74
	Group D	2.1	0.764	-1.66	5.86
Group C	Group A	-5.900*	0.001	-9.66	-2.14
	Group B	4.500*	0.012	0.74	8.26
	Group D	6.600*	<0.001	2.84	10.36

**Table 3:** Bonferroni Multiple Comparison Test to Assess Time Taken to Close the Flap (in Minutes) among the Four Groups

(I) group	(J) group	Mean Difference (I-J)	P	95% Confidence Interval	
				Lower Bound	Upper Bound
Group A	Group B	-10.500*	<0.001	-14.97	-6.03
	Group C	-19.300*	<0.001	-23.77	-14.83
	Group D	-12.800*	<0.001	-17.27	-8.33
Group B	Group A	10.500*	<0.001	6.03	14.97
	Group C	-8.800*	<0.001	-13.27	-4.33
	Group D	-2.3	0.955	-6.77	2.17
Group C	Group A	19.300*	<0.001	14.83	23.77
	Group B	8.800*	<0.001	4.33	13.27
	Group D	6.500*	0.002	2.03	10.97

Time required to raise the flap- Group A (42.90 ± 3.071 min) exhibited the longest mean time to raise the flap, followed by Group C (37 ± 3.399 min) and Group B (32.50 ± 1.650 min). Group D exhibited the shortest mean time to raise the flap (30.40 ± 3.534 min). The results of one-way ANOVA showed an overall significant difference among the groups. The results of Bonferroni multiple comparisons showed that the mean time required to raise the flap differed significantly between Groups A and B, Groups A and C, Groups A and D, Groups B and C, and Groups C and D (Table 2).

Time required for closure of flaps- Group C exhibited the longest time for flap closure (51.90 ± 4.53 min), followed by Group D (45.40 ± 3.68 min) and Group B (43.10 ± 3.38 min). Group A exhibited the shortest time for flap closure (32.60 ± 2.36 min). The one-way ANOVA results showed an overall significant difference among the groups. The results of Bonferroni multiple comparisons showed that the mean time for flap closure differed significantly between Groups A and B, Groups A and C, Groups A and D, Groups B and C, and Groups C and D (all P < 0.05); however, Groups B and D (P = 0.955) did not differ significantly (Table 3).

Accessibility to different levels of the neck nodes was assessed by a surgeon at the operation table. Group A had three patients exhibiting optimal access (30%), whereas Group C had the highest number of patients exhibiting optimal access to the lymph nodes (70%). Groups B and D had identical number of patients exhibiting optimal exposure of the neck.

Scar Evaluation- The Stony Brook Scar Evaluation Scale was used for the assessment of scar cosmesis. At discharge, mean scar evaluation scores differed significantly between Groups A and C and Groups C and D (P < 0.05). After 1 month, the scar evaluation scores were similar to those recorded at discharge. Mean scar evaluation scores differed significantly between Groups A and C and Groups C and D (P < 0.05). The scores did not differ significantly between the other groups. After 2 months, the mean scar evaluation scores differed significantly between Groups A and B, Groups A and C, and Groups C and D, whereas at the end of 3 months, mean scar evaluation scores differed significantly between Groups A and B, Groups A and C, and Groups C and D. 

Wound Healing- At discharge, wound healing was assessed. Five out of ten patients showed contraction of the wound in Group C (50%) with three of the five patients showing marginal necrosis at the triradiate area. Complications related to wound healing were minimal in Group A, where only one in 10 (10%) patients showed wound contraction. After 1 month, the contraction of the wound persisted in five of 10 patients in Group C. After 2 months, one patient in Group A presented with an infection in the neck region, whereas no fresh complications were noted in the patients of Groups C and D. After 3 months, the dehiscence in the Group C patients resolved without any intervention, whereas the contraction of wound reduced in two of the five patients who exhibited wound contraction. Contraction was persistent in one patient (10%) in Group B by the end of 3 months.

## Discussion

Surgical incisions are designed to follow specific anatomical landmarks (Patnaik et al., 2000; Tubach et al., 2013). The type of incision normally depends on the location of the primary lesion and the type of neck dissection planned (Tubach et al., 2013). A skin incision for neck dissection must have defined anatomical landmarks and should allow adequate exposure of the surgical site. It should ensure that the skin flaps are properly vascularized and should be repaired with ease. The incision should also be designed to provide protection to vital structures in the vicinity. The present study was undertaken to evaluate the commonly used incisions for radical neck dissection and determine the number of criteria met by each incision technique. This was expected to establish harmony between a surgeon’s perspective of adequate exposure of the surgical field and patients’ desire of postoperative cosmesis after a major ablative procedure. 

Intraoperative parameters were used to assess the time required to handle each of these flaps and incorporate the surgeon’s ease of performing the procedure. Because all the neck dissections were performed by the same surgeon, the chance of operator bias was eliminated. 

Time required to raise the flap through a particular incision was calculated from the time of placement of the incision using a BP blade to the commencement of nodal dissection. Statistical analysis of the collected data showed a highly significant difference in the time required between Groups A and B, Groups A and C, Groups A and D, and Groups C and D, whereas a significant difference was observed between Groups B and C (P = 0.012). Group A, comprising patients operated using the Macfee incision, exhibited a significantly longer time for raising the flap than the other Groups B, C, and D. This long time is attributable to the wide bridge present between both the transverse incisions and the cumbersome method of working under a tunnel. The same drawback has been highlighted by Macfee in his own study, where he considered the dissection more difficult and time consuming than vertical incisions (Macfee, 1960). Group C, comprising patients operated using the modified Schobinger incision, also showed a significant increase in time than Groups B and D. This observation can be explained by the need to raise the anterior and posterior parts of the flap along the vertical incision. Although raising parts of the flap lengthens the operation time, it provides excellent access to the surgical field.

The time required to close the flaps was also assessed intraoperatively. On statistical computation of data, a highly significant difference was observed between Groups A and B, Groups A and C, Groups A and D, Groups B and C, and Groups C and D. Flap closure in Group A (Macfee incision) required the shortest time all the groups (Mean = 32.60 min). Flap closure in Group C (modified Schobinger incision) required the longest time for closure (51.90 min). The use of the Macfee incision, which has only two parallel transverse incisions along the skin creases, thus reducing the operation time considerably. The reduction in closure time also compensates for the initial time required to raise the flap.

As stated by Gavilan and Herranz (2004), accessibility of all the levels of neck lymphatics is probably the most vital aspect of the planning of an incision. The surgeon assessed the exposure of the neck node levels during the procedure. The use of the Macfee incision provided adequate exposure of all the lymph node levels only in three of 10 patients (30%). In patients with a slightly shorter necks or patients with reduced elasticity of skin, the use of the Macfee incision was cumbersome because access to the posterior limits of the neck was difficult. By contrast, Group B, C, and D incisions provided good to fair exposure of the neck; the maximal exposure was observed in Group C (70%). Only one patient in Group D (reverse hockey-stick incision) exhibited poor access to the submandibular lymph nodes. However, poor access resulted from adherence of the node to the skin due to infiltration by the tumor, which did not allow adequate retraction for access to the region.

As stated by Popescu et al., (2012), the sacrifice of the internal jugular vein in a patient with head and neck cancer minimally affects quality of life. Transitory postoperative edema may be noted, which subsides in 1 week. However, unsuitable antidiuretic hormone secretion may cause edematous soft tissues after sacrifice of the internal jugular vein. Two patients among 40 patients had their internal jugular veins sacrificed due to infiltration of the tumor into the carotid sheath and its adherence to the vein. One of the patients belonged to Group B, whereas the other belonged to Group C. No complications were encountered during postoperative or follow-up periods in the two patients. Thoracic duct injury was observed in three patients in Group A and two patients each in Groups B and C; all injuries were on the left side. All the injuries occurred during dissection of Level IV lymph nodes. All the cases of duct injury were readily repaired on the operating table using silk sutures. None of the patients exhibited any persistent leaks postoperatively. The spinal accessory nerve was inadvertently injured in two patients, one each in Groups B and C. The nerve was injured in the patient from the modified Macfee incision group during the dissection of level IIa. In the other patient, the nerve was injured during removal of the sternocleidomastoid muscle. In both the patients, the exposure to the region of dissection was adequate from the surgeon’s perspective. The patients complained postoperatively of restricted shoulder movements.

Analysis of the statistical data showed a highly significant difference in the scar esthetics between the patients in the groups receiving the Macfee incision and the modified Schobinger incision and also between the patients in the groups receiving the modified Schobinger and reverse hockey-stick incision. The evaluation of scars after 1 month also exhibited the same results; significant differences were observed between Groups A and C and Groups C and D. After 2 and 3 months, respectively, a significant difference was observed in scar cosmesis between the Group A (Macfee incision) and Group B (modified Macfee incision); however, the the findings of the groups were comparable during discharge and at 1 month. The modified Macfee incision, which is placed by modifying the upper transverse incision of the classical Macfee incision by creating two near-vertical lines and then making a transverse incision, causes the formation of a relatively narrow bridge. This in turn provides excellent exposure of the neck node levels, but it compromises the highly esthetic outcomes of the classical Macfee incision. The difference can be satisfactorily explained by the placement of two near-vertical lines of the incision on the neck, which compromises the vasculature of the flap from both anterior and posterior directions. Notably, the mean score for scar cosmesis was the highest in Group A (Macfee incision) throughout 3 months of follow-up, thus validating Macfee’s view of producing inconspicuous scars in these patients (Macfee, 1960). The patients in Group D (reverse hockey-stick incision) also had highly inconspicuous and esthetically acceptable scars. The finding was consistent with the findings reported by Omura et al., (1999) who performed a long-term evaluation of scars and stated that the incision was satisfactorily hidden under the lower border of the mandible. Patients in Group C (modified Schobinger incision) had the lowest cosmesis scores over 3 months. It can be easily explained by the placement of a triradiate incision that reduces blood supply to the tripartite point and causes cosmetic compromise. The incision is also extended against the natural skin creases in a vertical direction, thus causing additionally unacceptable scars. This observation was contrary to the findings of a study by Guerrissi (2007) in which the use of the modified Schobinger incision was reported to cause inconspicuous and cosmetically acceptable scars.

Wound healing was assessed over 3 months in the patients based on marginal flap necrosis, dehiscence, contraction, infection and exposure of vessels. We did not encounter any cases of vessel exposure in the entire study population. A high incidence of marginal flap necrosis and dehiscence was noted in patients who received the modified Schobinger incision, around the triradiate point due to a considerable compromise in the blood supply. A similar finding was reported in a study done by Yii et al., (1999) who evaluated the use of the apron flap incision, modified Macfee incision, and the modified Schobinger incision. They also found a statistically significant difference in wound dehiscence between the apron flap incision and the Schobinger group. A significant increase in dehiscence was also observed in irradiated patients in the Schobinger group in the same study. A conservative management procedure was used for the marginal necrosis of the flaps because they were superficial, while the dehiscence margins were renewed under local anesthesia and reapproximated using 3-0 silk sutures. No dehiscence was observed in the patients of any group after 3 months, while marginal necrosis persisted in one patient of the Schobinger group. Infections of the incision sites were treated conservatively. 

Although the study comprised only 40 patients, it showed highly significant outcomes for the parameters that were recorded. This study is the first to measure the time required to raise and close the flaps by using different incisions and the effects of the time required on the overall duration of the surgery. The study has also incorporated four incisions, with different anatomical bases, to study their effects on the overall wound healing and cosmesis of the scar with a follow-up period of 3 months, which also yielded highly significant results.

The study has some technical and statistical limitations. No separate subgroups were assigned for patients undergoing primary closure of defects or with reconstruction, and the lack of separation into subgroups might have affected different parameters. The statistical data on wound healing could not be assessed using statistical tests because some of the groups did not exhibit complications. Hence, the significance could not be compared on statistical basis. Thus, a study of a similar design on a larger sample with a longer follow-up period is required.

The study found provided some highly significant results. Patients operated using the Macfee incision showed the optimal results in terms of wound healing and cosmesis of scar after 3 months. The Macfee incision also requires the least time during wound closure, thus compensating for the longest time required to raise it. The reverse hockey-stick incision proved to be the most versatile among the four incisions, showing a fine balance between exposure of the neck node levels, scar cosmesis, healing and the relatively less duration in raising and closure of the flap. The modified Macfee incision, modified Schobinger incision, and reverse hockey-stick incision can all thus be used to obtain adequate exposure of the lymphatics. Although the preference for cosmesis comes after clearance in case of oncologic resections, the Macfee incision and reverse hockey-stick incision are the incisions of choice if esthetics is most crucial. A similar study with larger sample size is warranted, with longer follow-up period to evaluate the long-term functional and esthetic outcomes of using these incisions. 

## Funding statement

No source of funding.

## Conflict of interest

Nil.
